# Copen-SCALE: scaling-up an effective intervention to childcare institutions in Copenhagen – a stepped wedge type II hybrid effectiveness-implementation trial. A study protocol

**DOI:** 10.1186/s12889-025-24463-9

**Published:** 2025-10-09

**Authors:** Mia Nyvang Stilling, Line Lindberg, Laura Downs Tuck, Ole Henning Sørensen, Andreas Holtermann, Charlotte Diana Nørregaard Rasmussen

**Affiliations:** The National Research Center for the Working Environment (NFA), Copenhagen, Denmark

**Keywords:** Childcare worker, Occupational health, Implementation, Scale-up, Incomplete stepped wedged cluster trial, Musculoskeletal pain, Sickness absence

## Abstract

**Background:**

This study protocol describes the evaluation of the scale-up of an effective intervention to potentially all 350 childcare institutions in Copenhagen. This project represents a unique opportunity previously unseen in occupational health and safety – namely the scale-up of an effective workplace intervention (TOY) to hundreds of workplaces. The aim of the project is to evaluate the scale-up of the TOY intervention, titled “Copen-SCALE.”

**Methods:**

The study is a 4-year type II hybrid stepped-wedge trial that will evaluate the effectiveness, cost-effectiveness, and implementation of an intervention across up to 350 childcare institutions in Copenhagen Municipality. The original TOY intervention included three participatory ergonomic workshops at childcare institutions, facilitated by external ergonomic consultants. The intervention had high feasibility and demonstrated a significant reduction in pain-related sickness absence among the childcare workers. Copen-SCALE’s target population now includes childcare workers in both day nurseries (ages 0–3) and pre-schools (ages 3–6), expanding beyond the original TOY RCT trial group. Moreover, there has been further development of the intervention by including best practices and research findings to the TOY concept, leading to the development of Copen-SCALE. Copen-SCALE includes four evidence-based components: child self-reliance, ergonomics, pain management education, and health-promoting activities. Copen-SCALE aims to include up to eighty-seven childcare institutions each year from 2024 to the end of 2027. Mixed methods will be employed to investigate effectiveness, cost-effectiveness, adaptation, and implementation of Copen-SCALE.

**Discussion:**

Scaling up effective interventions is crucial for achieving widespread benefits. Yet, this is underexplored in occupational health. The TOY intervention, being cost-effective and scalable, has the potential to contribute with valuable evidence, informing policies to enhance childcare workers’ health, well-being, and work environment. We anticipate that the findings from this project will inform wider dissemination of the intervention to childcare workers in more municipalities in Denmark.

**Trial registration:**

The study was prospectively registered in a clinical trial register (ISRCTN14831585). Registered 04 January 2024, ISRCTN - ISRCTN14831585.

**Supplementary information:**

The online version contains supplementary material available at 10.1186/s12889-025-24463-9.

## Background

Childcare serves as a fundamental social service and allows parents to participate in employment, education, and training, all the while ensuring the well-being and growth of their children in a stable and nurturing setting. The profession of childcare is however facing a labor shortage globally [1]. Danish childcare workers report high prevalence of musculoskeletal pain (38%), compromised mental well-being, and as much as 14 days annual sickness absence [2, 3]. The research literature points towards high physical and psychosocial work demands, musculoskeletal pain, and poor mental health and stress as main contributors to the high rates of sick leave and turnover among childcare workers [4, 5]. Consequently, there is a compelling need to ensure the vitality of childcare workers, enhancing their ability to care for children for more years.

Musculoskeletal pain is one of the biggest burdens of disease globally affecting approximately 1.71 billion people corresponding to about 149 million years lived with disability [6]. In Denmark, musculoskeletal pain represents the predominant cause for visits to general practitioners, hospital admissions, premature workforce attrition and sick leave episodes [7]. The associated annual costs incurred by the healthcare system of musculoskeletal pain are estimated to be 12 billion DKK, with an additional productivity loss of 40 billion DKK [7]. Demanding physical tasks at work contribute to a noteworthy proportion of low back pain (25%) and absenteeism (up to 40%) [8, 9]. Therefore, musculoskeletal pain puts a substantial toll on workplaces, highlighting the compelling rationale for workplaces to prevent musculoskeletal pain.

In the years 2018–2019, we conducted a randomized controlled intervention involving 190 childcare workers across sixteen childcare institutions in Copenhagen. The objective was to improve the capability of childcare workers in promoting self-reliance and learning among nursery children, incorporating ergonomic considerations, especially in physically demanding situations. This initiative was known as the TOY project [10]. The intervention had high feasibility and demonstrated a significant reduction in pain-related sickness absence among the childcare workers [11]. Moreover, the intervention had low implementation costs, and the return-on-investment of the intervention was 63%, indicating a monetary benefit for the childcare institutions [12].

Because of these findings, the Children and Youth Administration in Copenhagen Municipality opted to expand the TOY intervention. The scale-up initiative is set to encompass all 350 childcare institutions within the municipal area, with implementation scheduled to commence from the beginning of 2024 and extend through 2027. Participation will be voluntary. The TOY intervention was fundamentally a collaborative effort involving the participating workplaces as well as occupational health and safety representatives within the municipality (ergonomic consultants), thus ensuring its practical viability and efficacy. Importantly, the intervention was implemented under realistic conditions, involving the training of local implementers for intervention delivery.

Hence, the TOY intervention aligns with the concept of scalability, defined as “*the capacity of a health intervention proven efficacious within controlled settings or on a small scale to be extended into real-world scenarios*,* encompassing a more substantial portion of the eligible population while upholding its effectiveness*” [13]. These findings support that the TOY intervention is sufficiently cost-effective for scale-up to childcare institutions.

The original TOY intervention consisted of three participatory ergonomic workshops conducted at each childcare institution during regular staff meetings facilitated by an ergonomic consultant (an occupational therapist or a physiotherapist) from the Working Environment Consultancy Copenhagen.

Participants were briefed on the intervention’s primary focus, integrating participatory ergonomic action plans into their core tasks. The intervention, guided by a six-step participatory ergonomics framework inspired by Haines and colleagues [14], began with pain mapping and risk identification in the first workshop. Working teams then created and implemented action plans based on their findings. The second workshop involved evaluating the initial solutions and creating new action plans, while the third workshop assessed the new plans and discussed how to sustain changes [15]. Between workshops, childcare workers implemented 85% of suggested solutions during their working day [16].

Scaling up an intervention requires adaptations. In the process of scaling up TOY, a first adaptation has already been required. This is that the Copenhagen municipality has decided to expand the target population from childcare workers in day nurseries for children aged 0–3 (population in the TOY RCT trial) [10], to also include childcare workers in pre-schools for children aged 3–6 in Copen-SCALE. Moreover, since finalizing TOY in 2019, the Working Environment Consultancy Copenhagen have collaborated on other research projects and have added new best practices and research findings to the TOY concept, leading to the development of Copen-SCALE. In addition to the elements from TOY (ergonomics and children’s self-reliance and motor skills), Copen-SCALE also includes dimensions focusing on educating workers how to prevent and handle pain [17], as well as incorporating more health-promoting physical activities in their daily work routine [18]. Copen-SCALE thus consists of four evidence-based areas of intervention: (1) children’s self-reliance and motor skills, (2) ergonomics, (3) education in prevention and handling pain, and (4) health-promoting physical activity.

This study aims to evaluate the effectiveness, cost-effectiveness, implementation, and adaptation of the Copen-SCALE intervention in up to 350 childcare institutions within Copenhagen municipality. This will be completed in a type II hybrid effectiveness-implementation trial with several studies: The first two studies, an effectiveness evaluation and economic evaluation, will be conducted utilizing an incomplete stepped-wedge controlled trial [19] with the primary outcome of pain-related sickness absence. The next study is an evaluation of the required adaptations to the TOY intervention to enable the scale-up. This evaluation will consider changes at the interventional level as well as the institutional level. The fourth study is an implementation evaluation focusing on the following outcomes: 1) acceptability, (2) adoption, (3) appropriateness, (4) cost, (5) feasibility, (6) fidelity, (7) penetration, and (8) sustainability.

We anticipate that the findings from this project will inform wider dissemination of the intervention to childcare workers in Denmark.

## Methods

### Data protection, ethical approvals, and trial registration

The National Research Center for the Working Environment has an institutional agreement with the Danish Data Protection Agency about procedures to treat confidential data (journal number 2015-41-4232). The Danish National Committee on Biomedical Research Ethics (the local ethics committee of Frederiksberg and Copenhagen) has reviewed the study description and determined that according to Danish law it is not necessary to seek formal approval (H-23049692). Written informed consent to participate in this study will be provided by the participants. Moreover, the study was prospectively registered in a clinical trial register (ISRCTN14831585). The study is reported according to The StaRI standard checklist [20].

### Setting and participants

All 350 childcare institutions in the municipality of Copenhagen are invited to be enrolled in Copen-SCALE. All childcare workers (~ 7.200) [21] in the 350 childcare institutions will be eligible for participation in the evaluation. Copen-SCALE is planned to have a duration of 4 years, including up to eighty-seven childcare institutions per year, beginning in January 2024 and ending ultimo 2027.

### The intervention

**Fig. 1:**
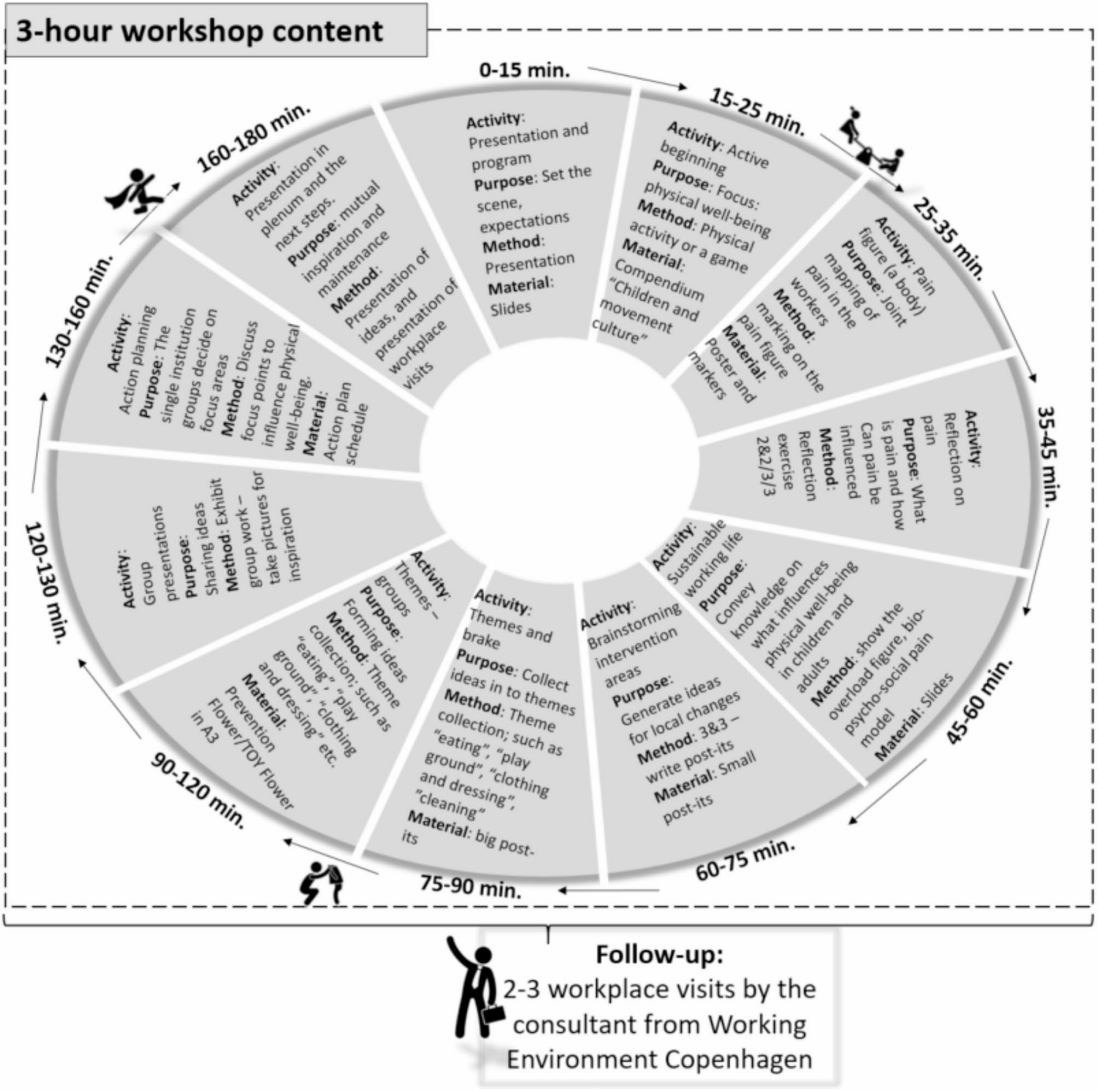
Intervention model for Copen-SCALE. Copen-SCALE consists of four evidence-based components. The intervention begins with a three-hour workshop during working hours, educating childcare workers in key areas within the four intervention components, and prompting them to identify work-related challenges and develop action plans. Subsequently, two to three consultant visits occur over a period of 3–6 months to provide ongoing support and monitor implementation for sustainability

Copen-SCALE consists of four evidence-based areas of intervention: (1) children’s self-reliance and motor skills, (2) ergonomics, (3) education in prevention and handling pain, and (4) health-promoting physical activity. The intervention involves a structured 3-hour workshop with employees during working hours, cf. Figure 1. This workshop focuses on educating childcare workers about the intervention components while also encouraging them to identify specific work-related challenges they wish to address. Additionally, participants develop actionable plans to improve their work situations. Following the workshop, the intervention includes 2 to 3 visits by a consultant at the workplaces. During these visits, the consultant provides ongoing support and monitors the implementation of the intervention to ensure its effectiveness and sustainability.

### Implementation strategies

Copenhagen Municipality has chosen to scale up the adapted TOY intervention to all childcare institutions, giving rise to the Copen-SCALE initiative. The municipality has formulated a business plan, securing funding for delivery and implementation of the Copen-SCALE implementation over the forthcoming four years. The childcare institutions involved will receive compensation for the time dedicated to implementing the intervention. The intervention will be implemented over 6–8 months in each individual institution, and as in TOY, consultants from the Working Environment Consultancy Copenhagen will deliver the intervention in Copen-SCALE. The Working Environment Consultancy Copenhagen specializes in workplace ergonomics and has previous experience with workplace health and safety research projects.

## Effectiveness evaluation

### Study design

**Fig. 2:**
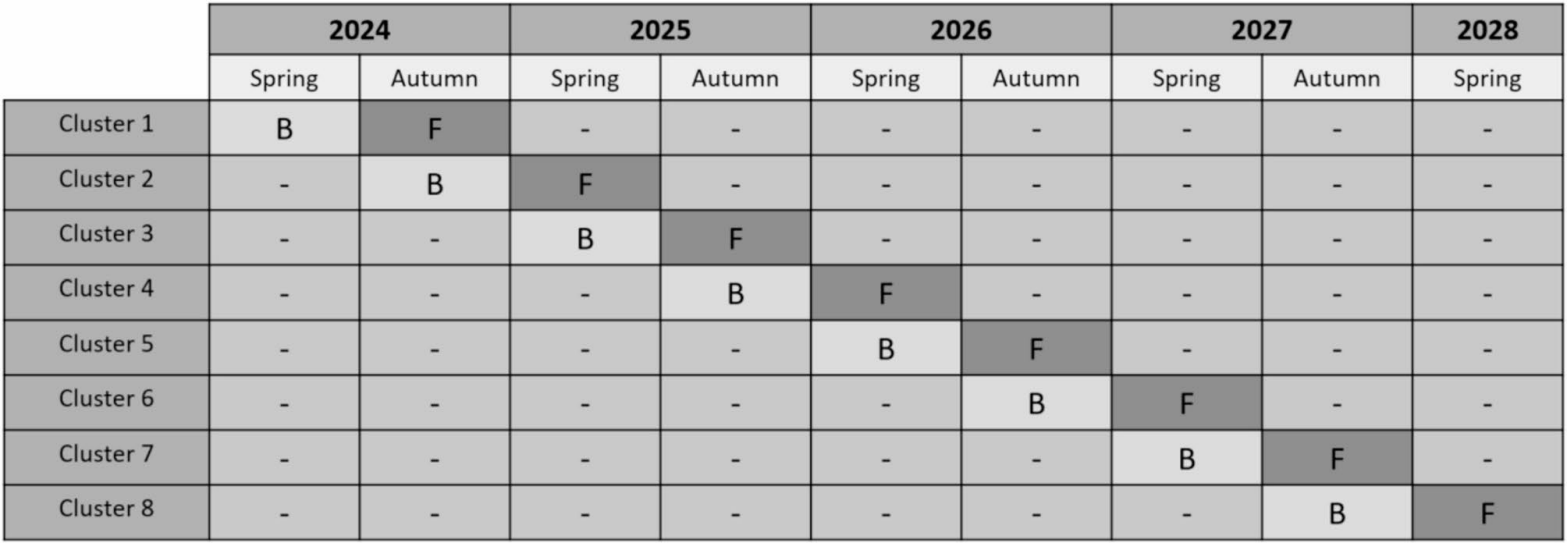
The incomplete non-randomized stepped-wedge design. The institutions within each cluster made up by time periods (spring/autumn) will be measured before the intervention is implemented (at baseline = B) and then after 6–8 months (at follow-up = F). For practical reasons, the institutions within each cluster will start the intervention at different time points within the 6-month intervention period. When a cluster completes the intervention and has their follow-up measurement, the baseline measurement of the next cluster will function as a control

This is a 4-year prospective type II hybrid stepped-wedge controlled trial evaluating effectiveness and cost-effectiveness outcomes along with an implementation evaluation [22]. The gold standard for an intervention study is usually a randomized controlled trial (RCT). Randomization is not always feasible when conducting scale-up studies because of the dynamic context in which they occur [19, 23, 24]. Hence, this intervention utilizes a pragmatic stepped-wedge controlled trial design, a methodology that has exhibited success in prior workplace studies [10, 11, 25]. A stepped wedge controlled trial assesses an intervention rolled out in stages over multiple periods. There is considerable variety in the design and conduct of stepped wedge controlled trials. One modification is the incomplete cross-sectional stepped-wedge cluster-controlled trial, where not every cluster is measured at every time point (17). The incomplete cross-sectional stepped-wedge cluster-controlled trial fits well with the planned implementation of the 350 childcare institutions over the course of 4 years, equivalent to about forty-four institutions per sixth month intervention period. The clusters will be formed based on the preferences of childcare institutions, and not by randomization. Randomization was not possible due to the restrictions from the Copenhagen Municipality in how to implement the intervention. The non-randomization is a limitation, but we will adjust for potential confounders in the analyses (see later). The design is illustrated in Fig. 2. A cluster consists of the workplaces that begin the intervention within a given period (spring or autumn). The baseline measurements from clusters prior to implementing the intervention will function as controls for the previous cluster who has completed the intervention. Follow-up measurements (F) will then be compared to baseline measurements (B) and serve as a control (Fig. 2).

### Sample size

It is important to consider statistical power when designing an implementation study. As implementation research is aimed at system-level changes, the power of a given study depends on working units (e.g., childcare institutions) as opposed to individual employees. Therefore, traditional methods of sample size and power calculation are usually not applicable as they fail to consider clustering and the extended time-frames of implementation studies [26]. To address this complexity, we utilize a recommended tool, the Optimal Design with Empirical Information (OD+), for effectively planning and powering these complex study designs [27]. Considering a power of 80%, an alpha level of 0.05, and a reliability of 0.70, the required sample size for our study is determined to be seventy-four childcare institutions. Given that we have up to 350 eligible childcare institutions for participation, we believe that we will be able to fulfill the targeted number in our sample size calculation.

### Data collection

Data collection will primarily be distributed to the childcare workers through a weblink on AULA (A digital platform used by childcare institutions in Denmark for communication within the institution) that directs them to the electronic questionnaire (SurveyXact). Data will be collected for all childcare workers at baseline and after the intervention period (approximately 6–8 months). To ensure data integrity, a blind assessor will check each variable for *out of range* or *implausible values*. Once all data are checked, we will import the dataset in panel format, into RStudio.

To ensure data integrity, a blind assessor will check each variable for *out of range* or *implausible values*. Once all data are checked, we will import the dataset in panel format, into RStudio.

To describe the study population, we will collect data on the childcare workers including sociodemographic information (i.e., age, sex, and work-related factors such as job title, seniority, weekly working hours), work environment factors, and psychosocial work environment factors measured using questions from the Danish Psychosocial Questionnaire [28]. Moreover, information about each childcare institution (e.g., type of institution (public or private), number of units, and number of employees) will be collected from registers from the municipality.

### Effeciveness outcomes

With inspiration from Proctor et al., we have divided the outcomes into implementation outcomes (see later), organizational and individual outcomes [29]. Thus, the effectiveness outcomes are measured as organizational outcomes, individual outcomes (employees).

### Organizational outcomes

At the organizational level, we will use the workplace registrations for sickness absence (absenteism). Presenteism will be measured by questionnaire at the employee level [30, 31]. Together, this information will be used for the cost-effectiveness analysis from the perspective of the employer (described later).

### Individual outcomes

At the individual participant level, we will collect information from questionnaires using validated questions. The main focus will be pain-related sickness absence [32], musculoskeletal pain (location, duration and intensity) [33, 34], work-related pain interference [35], self-perceived physical activity literacy [36], fear avoidance [37], physical exertion and pain-related occupational health literacy [17].

### Effectiveness analysis

Evaluation of effectiveness of the intervention will be performed using multilevel analyses using pooled individual level data. Multilevel analyses take clustering of observations of workers within the same team into account [38]. All analyses will be performed according to the intention-to-treat principle, including all eligible participants without imputations because mixed models inherently account for missing values [39]. In this analysis, the intervention will be modeled as a fixed effect and participants will be entered in the model as a random effect nested in clusters to account for the cluster design. Time will be treated as a dummy-coded categorical variable, and we will examine group × time interactions to determine intervention effects. In addition, we will take into account the possible differences between the groups at baseline [40]. Moreover, we will adjust for possible confounders at both individual and organizational level due to the non-randomization.

The mixed model analysis requires assumptions about the data. These include independence of observations, normality, and heteroscedasticity. Moreover, we will also consider other distributions of data and use the most appropriate statistical model for the collected data. Missing data are assumed to be Missing at Random. Missing at Random describes the probability of a missing observation being independent of prior observations, but conditional on other observed values. Repeated measures analysis also assumes a correlation structure between the repeated measurements. The correlations can be fixed (structured model), decaying (autoregressive model) or unconstrained (unstructured model). We will check the correlations between the repeated assessments and use the appropriate correlations according to the best model fit (tested by likelihood ratio test) [38–40].

### Cost-effectiveness evaluation

An economic analysis of Copen-SCALE will be conducted to inform decision makers about the costs and benefits of implementing Copen-SCALE. Two types of economic evaluation (i.e., cost-effectiveness and cost-benefit analysis) will be performed, both from employers’ perspective. All analyses will be based on data collected between baseline and 6–8 month follow-up, according to the intention-to-treat principle with multiple imputation of missing data [39].

## Data collection for the economic evaluation

### Health-related productivity loss costs

Absenteeism (days missed from work due to sickness absence) will be measured from workplace registers.

Presenteeism (reduced performance related to health problems while being present at work) will be measured by a small electronic questionnaire for employees at baseline and follow up [30, 31]. Absenteeism and presenteeism costs will be estimated using the Human Capital Approach, because this method assumes that the salary of an employee equals the productivity loss of that employee. As a result, health-related productivity losses (i.e., both presenteeism and absenteeism) will be valued using average gross salaries in the municipality depending on personnel category. To assess the robustness of the Human Capital approach we will supplement with a Friction-Cost approach.

### Intervention costs

We consider the following costs for implementing the intervention:

1. Staff time: Employees’ and managers’ participation in intervention activities (registration of participation in activities that are not directly part of daily work (e.g., a workshop/meeting for planning the activities). Employees’ and managers’ costs will subsequently be valued based on their average gross salaries in the municipality, including overhead costs.
2. Consultants’ time: The time spent on implementing the intervention by the consultants will be registered. Their costs will be based on their usual consultancy fees.
3. Consumables: Materials cost, i.e., exercise bands and activity cards, will be assessed using invoices.
4. Overhead: We incorporate an overhead cost of 20% of the intervention (such as the cost of booking the meeting rooms, telephone bills, catering, electronics usage).

### Cost-effectiveness analysis

The total employer’s costs of the intervention will be estimated and compared between the intervention and control group. The incremental cost-effectiveness ratio (ICER) will be calculated by dividing the mean difference in costs (incremental cost) between both groups by the difference in effects (incremental effect) on the outcome measures, i.e., pain-related sickness absence and musculoskeletal pain. The 95% confidence intervals for the ICERs will be estimated using the bootstrapping method (1000 bootstrap samples with replacement) [41]. The bootstrapped incremental cost and effect pairs will be graphically presented on cost-effectiveness planes [42]. Cost-effectiveness acceptability curves will be estimated for the probability that the intervention will be more cost-effective than usual practice as a function of the employer’s willingness to pay.

### Cost-benefit analysis

In a cost-benefit analysis, we will calculate the net benefit of the intervention, expressed as Return On Investment (ROI) and Benefit to Cost Ratio (BCR). The costs comprise all the relevant expenses for the intervention, detailed above. The benefits comprise the monetary gain due to a change in absenteeism and presentism. Further, to arrive at a conservative estimate we will also conduct a separate cost-benefit analysis using only the change in sickness days, absenteeism, reported by institutions and/or the municipality. To simulate potential incremental cost-benefit gained in the years after the intervention we will simulate two scenarios. In the first, we assume a constant effect of the intervention over time. In the second scenario, we assume a fade-out effect in which the effect fades-out over a 5-year period with a constant rate.

To supplement the cost-benefit analysis, we perform a cost-effectiveness analysis considering also changes in experienced pain. The costs are calculated as intervention costs minus sickness-related costs. The effects comprise musculoskeletal pain changes, using questionnaire data on average pain over the last seven days, viewed as an interval scale. The 95% confidence intervals for the ICERs will be estimated using the bootstrapping method (one thousand bootstrap samples with replacement) (71). The bootstrapped incremental cost and effect pairs will be graphically presented on cost-effectiveness planes (72). Cost-effectiveness acceptability curves will be estimated to assess the probability of the intervention being cost-effective as a function of the employer’s willingness to pay.

### Implementation evaluation

We will employ the taxonomy of implementation outcomes outlined by Proctor et al. (2011) - Implementation Outcomes Framework (IOF) [29]. Implementation will be evaluated through the outcomes of acceptability, adoption, appropriateness, feasibility, fidelity, implementation cost, penetration, and sustainability, each representing a distinct dimension of the implementation process (Table 1).

**Table 1:** Implementation outcomes: inspiration from Guerin et al. 2022 [44] which will be used to evaluate how and to what extent Copen-SCALE has been implemented across the participating childcare institutions across Copenhagen municipality

Outcome	Definition	Measure or scale
PRIMARY IMPLEMENTATION OUTCOME		
Fidelity	*Degree to which the intervention is implemented as intended by the consultants.*	-Interventions logs filled in by the consultants - Observations of intervention activities
SECONDARY IMPLEMENTATION OUTCOMES		
Acceptability	*The perception among childcare institutions that the intervention is agreeable and satisfactory.*	- Questionnaire: Acceptability of Intervention Measure (AIM) [43]
Adoption	*Intention among childcare institutions to employ the intervention (i.e.*,* “uptake”).*	- Recruitment data on workplace settings and characteristics
Appropriateness	*Perceived fit of the intervention for childcare institutions*	-Questionnaire: Intervention Appropriateness Measure (IAM) [43]
Costs	*Directly measured non-research costs*,* including all costs of implementation*	-Register of costs involved in implementing of the intervention
Feasibility	*Extent to which the intervention can be successfully applied to the childcare institutions*	-Questionnaire: Feasibility of Intervention Measure (FIM) [43]
Penetration	*Extent of integration of Copen-SCALE in the childcare institutions*	-Interviews
Sustainability	*Extent to which the intervention is maintained or institutionalized within the childcare institutions*	-Interviews

### Data collection

We will conduct a mixed method, multi-dimensional evaluation of the implementation of the Copen-SCALE. The evaluation will combine recruitment data, interviews, observations, and questionnaires. Table 1 outlines the implementation outcomes, their definitions and the assessment measure or scale.

### Primary implementation outcome - fidelity assessment

The fidelity assessment is inspired by the framework developed by Ferm et al. that suggests that fidelity includes information about the content of the intervention (success criteria), and quality of the intervention [45]. Questions about success criteria for specific activities in the Copen-SCALE intervention will be generated (see Table 2). After each activity, consultants will complete an intervention log containing two types of assessments:

**Table 2:** The fidelity questions that will be used to assess if the Copen-SCALE was successfully delivered as intended. These questions will be included in the intervention log that consultants will complete after each intervention activity is conducted. The consultants’ answers to these questions will be converted into a score from 0-100, where 100 indicates 100% fidelity [45]

PRIMARY INTERVENTION OUTCOME: FIDELITY	
Outcome	Measure
Content (success criteria)	Have you implemented the following according to the manual: [# success criteria]? Response: [“not implemented,” “partly implemented,” “completely implemented”, and “implemented more in depth.”]
Quality (Contribution to the participants’ learning)	Regarding today, to which extent have you contributed to… …the participants’ commitment and motivation? …ensuring the employees’ participation in the activity? …adapting the activity to the needs of the participants? …maintaining the participants’ attention? Response: [to a very large extent/to a large extent/some-what/to a small extent/to a very small extent]
Quality (Self-rated performance)	Suppose that your performance, at its best, is equal to 10 points. How would you rate your performance today?

Quality of Performance: Consultants will use a Likert scale to rate their own performance, with the prompt: “Imagine your performance can be worth ten points at its best. To what extent have you today contributed to engaging employees, ensuring their participation, adapting the program, or maintaining focus?” The score will later be converted to a scale from 0 to 100.

Content Fidelity: A yes/no scale will be used to evaluate whether the activity adhered to the protocol. If the response is “no,” consultants will have the opportunity to provide additional details explaining the deviation. Simple T-test analyses will be used to assess differences between the workplaces. Moreover, the research team will conduct random observations at the workplaces to further assess the fidelity of the intervention activities. An observation protocol will be developed to capture various aspects of the intervention activities, including information about content, quality, and contextual factors. The data gathered through this protocol will complement the information recorded in the consultants’ intervention logs, offering a more comprehensive understanding of the fidelity and contextual factors influencing the intervention’s implementation.

### Evaluation of adaptation

To assess the adaptation, we will (1) systematically record modifications made from the TOY intervention to the Copen-SCALE intervention, and (2) scrutinize the nature, extent, and rationale underlying these identified adaptations.

The Framework for Reporting Adaptations and Modifications-Enhanced to Implementation Strategies (FRAME-IS) [18] provides a systematic approach to documenting and analyzing adaptations made to interventions during implementation. This framework will guide the process of collecting, analyzing, and reporting data on intervention adaptations, fostering a comprehensive understanding of how adaptations impact the intervention’s effectiveness. FRAME-IS consists of seven modules (four core modules and three optional ones). The core modules cover describing the intervention and implementation strategies, as well as what modifications have been made, and why they have been made including the rationale for change. The optional modules define when the modification occurred and if it was planned along with who made the modification and how wide spread it is [46]. Additionally, Best Fit Framework Synthesis (BFFS) [19] will be used to allow the combination of FRAME-IS with another framework, ensuring that elements of adaptation not addressed by FRAME-IS are also captured.

**Fig. 3:**
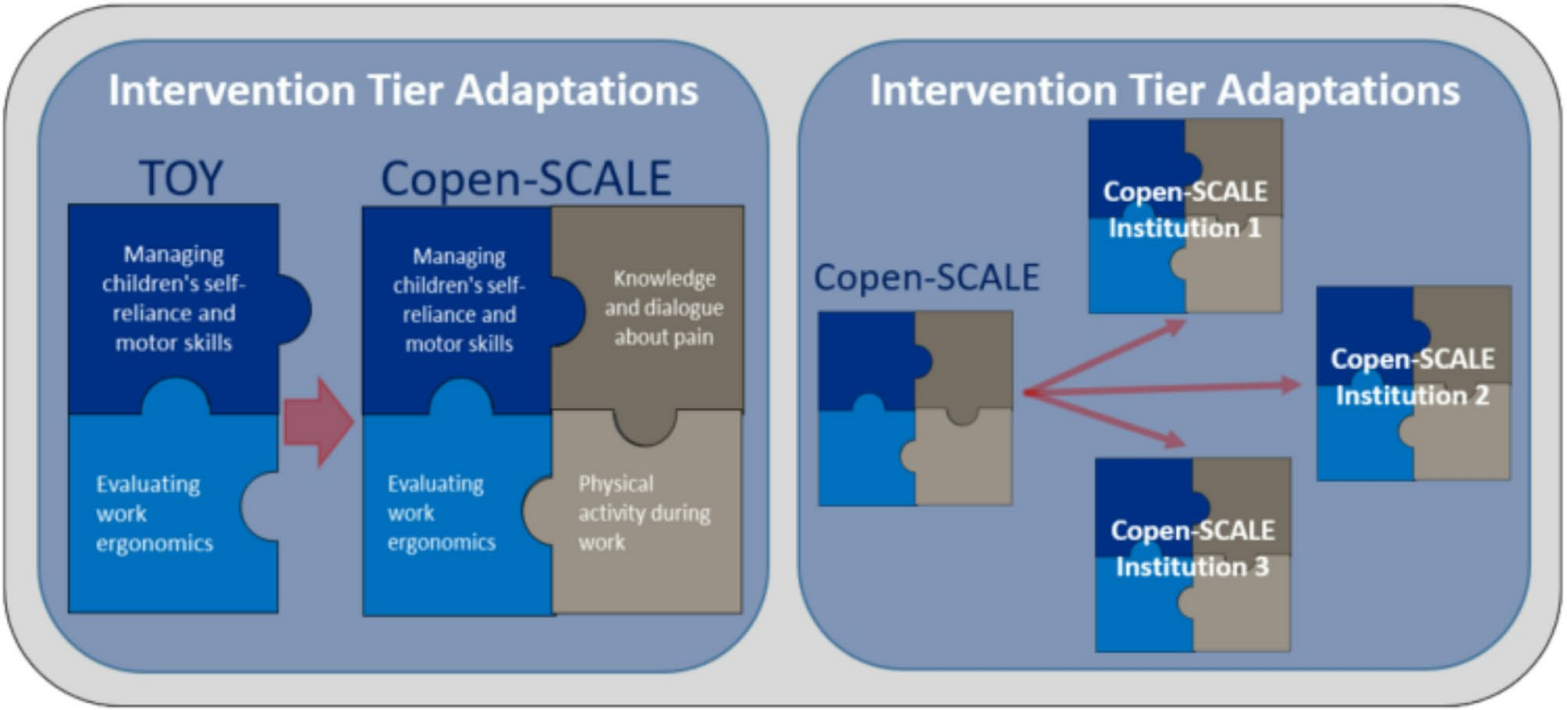
The two distinct tiers of adaptation: We will assess adaptations across both tiers. Firstly, adaptations are expected to occur as the TOY intervention transitions to the Copen-SCALE intervention. These adaptations could occur to content, target group, and methods of delivery and implementation as depicted. Secondly, given that the intervention consists of several components, it is expected that the delivery of the Copen-SCALE intervention will result in slight adaptations to the intervention to institutions’ individual needs and contexts. The institution tier adaptations are depicted here as implementation of Copen-SCALE in different institutions

The adaptation process manifests across two distinct tiers. Primarily, it is anticipated that adaptations will arise during the transition from TOY to Copen-SCALE and at the interventional level. Although a subset of these adaptations has been identified in advance, the Best Fit Framework Synthesis method will be employed to systematically assess all modifications made during this transition [47]. Furthermore, at each childcare institution, further adaptations are expected to take place as the intervention adapts to the needs, interests, and contextual nuances of each workplace. These tiers of adaptation are illustrated in Fig. 3.

### Data collection

The investigation of the adaptations that have occurred at the interventional level will be based on document analysis comparing the handbook from TOY to Copen-SCALE. At the institutional level, a combination of qualitative methods, such as focus groups and observations will be combined with document analysis to evaluate the adaptations. Data saturation will determine when qualitative data collection ceases.

### Interviews

We will conduct semi-structured focus group interviews with the consultants involved in the intervention’s implementation every six months. The primary emphasis will be placed on adaptation and fidelity at the institutional level, although certain elements of overall intervention development may also be addressed. We also conduct interviews with managers and employees involved in local implementation.

### Observations

During observations of intervention activities at childcare institutions, researchers will observe adaptations that have occurred.

### Document analysis

We will review documents related to the intervention, including intervention manuals, intervention presentations, meeting minutes, and communication records, to identify documented adaptations.

### Data analysis

Qualitative analysis will identify the types, contexts, reasons, and effects of intervention adaptations using codes from relevant frameworks. A coding manual will be applied to the interview and observation data, as well as during the document analysis using an abductive approach [48], where unexpected themes we be included as emerging codes.

## Discussion

This study protocol describes the evaluation of the scale-up of an effective intervention (TOY) to potentially all childcare institutions in Copenhagen (Copen-SCALE). This study presents a unique opportunity previously unseen in occupational health and safety in Denmark – namely the scale-up of the effective workplace intervention (TOY) to hundreds of workplaces. Scaling up effective evidence-based workplace interventions is vital for realizing population-wide benefits [13]. The concept of scale-up entails *“deliberate efforts to amplify the impact of proven health interventions*,* enabling them to reach a larger population and fostering lasting policy and program development”* [49]. Despite the demonstrated effectiveness of some workplace interventions in promoting health, their scale-up, dissemination and widespread adoption are often limited, hampering their potential for having societal impact.

The notion of scale-up has long been present in public health [13, 23]. In Denmark, the exploration of for example football exercise and health in research has evolved, ranging from modest to medium-sized randomized controlled trials (RCTs) targeting disease prevention, treatment, and health promotion. These efforts have evolved into extensive large-scale multi-centre projects [50]. Remarkably, to our knowledge, there are no successful interventions from a research study that have been scaled up within occupational health in Denmark and evaluated accordingly.

An international example of a successful occupational health intervention is the BeUpstanding Program, which expands on the “Stand Up Australia” project. The “Stand Up Australia” intervention was found to be effective at reducing workplace and overall daily sitting time in both the short term and the long term [51]. Thus, it was scaled up to more states in Australia. While the core components of the original intervention remained, adaptations were necessary to ensure feasibility and consistency with best practice. The adaptations included both changes in content and delivery [52]. While the BeUpstanding program’s intervention successfully penetrated diverse industries through online dissemination, publications, word of mouth, and presentations, challenges arose as few workplaces implemented the intervention as intended, consequently limiting its health-promoting impact [53]. The initial phase of the BeUpstanding study, which examines early adopters, and evaluates the program in the “real-world,” allowed for method improvement before extensive rollout. This approach is crucial to ensure the feasibility of broader implementation, as well as cost-effectiveness —yet, it is frequently overlooked [54, 55]. These findings highlight the difficulties in scaling up successful workplace interventions and emphasize the necessity for a phased implementation approach.

Scaling up effective interventions is essential for improving occupational health of childcare workers. The TOY intervention in Copenhagen Municipality is relevant, easy to implement, and cost-effective. With few successful examples of scaled-up workplace interventions, this study fills a significant evidence gap, contributing nationally and internationally.

Moreover, this project innovatively integrates dissemination and implementation research principles into scaling up the effective TOY intervention across hundreds of workplaces in Denmark. This approach enhances understanding of work environment factors and knowledge transfer processes, facilitating widespread improvements in childcare workers’ working conditions and health.

The findings from this research will inform policy formulation, leading to improvements in the working environment and overall well-being of childcare workers.

## Supplementary information

Below is the link to the electronic supplementary material.

ESM 1 (DOCX 80.1 KB)

## Data Availability

No datasets were generated or analysed during the current study.
